# Risk of Type 2 Diabetes According to Body Mass Index, Television Viewing Time, and Their Combination in the Japanese Population: Findings From the Japan Collaborative Cohort Study

**DOI:** 10.1155/jobe/1830356

**Published:** 2026-06-17

**Authors:** Shigekazu Ukawa, Wenjing Zhao, Satoyo Ikehara, Kokoro Shirai, Hiroyasu Iso, Akiko Tamakoshi

**Affiliations:** ^1^ Osaka Metropolitan University Graduate School of Human Life and Ecology, Osaka, Japan; ^2^ School of Public Health and Emergency Management, Southern University of Science and Technology, Shenzhen, China, sustc.edu.cn; ^3^ Department of Epidemiology and Behavioral Science, Faculty of Medicine, University of the Ryukyus, Okinawa, Japan, u-ryukyu.ac.jp; ^4^ Department of Social and Behavioral Medicine, Division of Health Science, Graduate School of Medicine, The University of Osaka, Osaka, Japan, osaka-u.ac.jp; ^5^ Institute for Global Health Policy Research, Bureau of International Health Cooperation, Japan Institute for Health Security, Tokyo, Japan; ^6^ Department of Public Health, Faculty of Medicine, Hokkaido University, Hokkaido, Japan, hokudai.ac.jp

**Keywords:** diabetes mellitus, obesity, overweight, prospective cohort study, sedentary behavior

## Abstract

**Objective:**

This study aimed to investigate the associations of body mass index (BMI), television viewing time, and their combination with the risk of Type 2 diabetes among Japanese individuals using data from a large Japanese prospective cohort study.

**Methods:**

This prospective cohort study included 23,604 Japanese participants (9,284 males and 14,320 females) aged 40–79 years. Baseline data were collected between 1988 and 1990 using a self‐administered questionnaire. In the 5‐year follow‐up survey, Type 2 diabetes was identified through self‐reported physician diagnoses. We used a logistic regression model to analyze the associations of BMI and television viewing time, and their combination with the risk of Type 2 diabetes.

**Results:**

During the 5‐year follow‐up, 5.9% of participants developed incident Type 2 diabetes. BMI and prolonged TV viewing time were independently associated with an increased risk of developing Type 2 diabetes. Individuals with obesity (BMI ≥ 30 kg/m^2^) who viewed TV for ≥ 7 h had a twentyfold increase in diabetes risk (odds ratio: 21.42, 95% confidence interval: 7.23–63.49) compared with those with normal BMI (18.5–24.9 kg/m^2^) who viewed TV for ≤ 3 h, indicating a significant interaction.

**Conclusions:**

Our study underscores the importance of addressing obesity and sedentary behavior to mitigate Type 2 diabetes risk.

## 1. Introduction

The prevalence of Type 2 diabetes is increasing rapidly worldwide, posing a major public health crisis. In 2021, 529 million individuals (6.1% of the global population) were living with the disease [1]. Globally, overweight and obesity are crucial factors in the development of Type 2 diabetes [2, 3]. Research in the fields of overweight/obesity and diabetes has consistently shown that being overweight doubles the risk of developing Type 2 diabetes, while obesity increases the risk by three to six times [4–6].

A comprehensive meta‐analysis highlights the association between prolonged television (TV) viewing, sedentary behavior, and an increased risk of Type 2 diabetes. After examining four prospective cohort studies, researchers found that two additional hours of daily TV viewing correlated with a 20% higher risk of diabetes [7]. Furthermore, extended TV viewing, which is linked to decreased physical activity and the consumption of unhealthy, high‐calorie foods, may contribute to weight gain [8]. However, the combined impact of overweight/obesity and TV viewing time on Type 2 diabetes has not been extensively reported, and limited evidence is available from Asian populations, where body composition and obesity distribution differ from those of Western populations [6]. TV viewing remains one of the most common forms of leisure‐time sedentary behavior and serves as a practical indicator of prolonged sitting in epidemiological studies. Few large‐scale prospective studies have evaluated the interaction between body mass index (BMI) and TV viewing time in relation to diabetes risk. Therefore, this study aimed to investigate the associations between BMI, TV viewing time, and the risk of Type 2 diabetes incidence among Japanese adults using data from a large prospective cohort study.

## 2. Methods

### 2.1. Study Population

**FIGURE 1:**
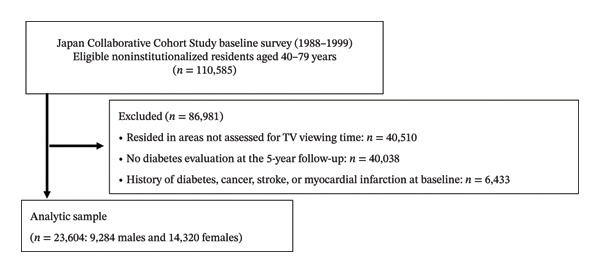
Flow diagram of participant selection.

The baseline survey of the Japan Collaborative Cohort Study conducted from 1988 to 1990 included 110,585 noninstitutionalized residents aged 40–79 years from 45 areas across Japan (46,395 males and 64,190 females) [9]. Participants were primarily recruited during health checkups conducted through household surveys. The participants completed a self‐administered questionnaire at baseline, with a high response rate (83%) [10]. Participants were excluded if they resided in areas not assessed for TV viewing time (*n* = 40,510); if diabetes evaluations were not conducted after a 5‐year follow‐up period (*n* = 40,038); or if they had a history of diabetes, cancer, stroke, or myocardial infarction at baseline (*n* = 6433). The final sample comprised 23,604 participants (9284 males and 14,320 females) (Figure 1). Individual informed consent was obtained before participation in 36 of the 45 study areas, including written informed consent in 35 areas and oral consent in 1 area. In the remaining 9 areas, group consent was obtained from the respective area leaders. These consent procedures were conducted in accordance with the applicable local regulations in each study area at the time of participant recruitment. The study protocol, including these consent procedures, was approved by the Ethics Committee of Hokkaido University (approval no. 14‐044).

### 2.2. Baseline Survey

Baseline data were collected through a self‐administered questionnaire on demographic characteristics, medical history, and lifestyle habits, as well as self‐reported height and weight. Average daily calorie intake was estimated using responses from a validated food frequency questionnaire administered at baseline. Participants reported habitual intake frequencies of commonly consumed foods, and daily energy intake was calculated using standard Japanese food composition tables, as described previously in the Japan Collaborative Cohort Study [11]. BMI was calculated by dividing the self‐reported weight in kilograms by the square of the self‐reported height in meters. BMI was then categorized into four groups: < 18.5, 18.5–24.9, 25.0–29.9, and ≥ 30.0 kg/m^2^ [12]. We obtained information on the average daily time spent viewing TV during the past year from the baseline survey question, “On average, how many hours do you watch TV?” Subsequently, TV viewing times were classified into four categories: < 3, 3–4.9, 5–6.9, and ≥ 7 h/day [13].

### 2.3. Ascertainment of Diabetes

In the 5‐year follow‐up survey, Type 2 diabetes was identified through self‐reported physician‐diagnosed cases. To evaluate the validity of these self‐reports, we compared them with the glucose levels or treatments in 1230 males and 1837 females. Diabetes was defined by a fasting serum glucose level of ≥ 7.8 mmol/L (≥ 140 mg/dL), a random glucose level of ≥ 11.1 mmol/L (≥ 200 mg/dL), or treatment with oral hypoglycemic agents or insulin. The sensitivity and specificity of self‐reporting are 70% and 95% for males and 75% and 98% for females, respectively [14].

### 2.4. Statistical Analysis

The participants’ baseline characteristics are presented as means ± standard deviations or percentages. We employed a logistic regression model to calculate the multivariable odds ratios (ORs) and confidence intervals (CIs) for the association between BMI, average daily TV viewing time, and incident Type 2 diabetes. This model was adjusted for potential confounders, with initial adjustments including demographic information such as age (treated as a continuous variable) and sex (categorized as male or female) in the first multivariable model. Subsequent adjustments incorporated educational level, employment status, smoking habits, drinking habits (categorized as never, former, current with 1–22 g/day, or current with ≥ 23 g/day of ethanol intake), daily sleep duration, exercise habits, perceived mental stress, and average daily calorie intake, all of which were included in the second model. The third model further integrated BMI and average daily TV viewing time. Additionally, we estimated the ORs for the combined effect of BMI and average daily TV viewing time on Type 2 diabetes incidence over a 5‐year follow‐up period. Statistical interactions were examined using the cross‐product terms, BMI, and average daily TV viewing time. *p* values for trends were calculated to assess linear associations between the four categories of BMI/average daily TV viewing time and Type 2 diabetes incidence. To treat incomplete data, we used multiple imputations using chained equations [15] to impute missing data. Specifically, 20 imputed datasets were created using the “mice” package in *R*. Analyses were then conducted separately in each imputed dataset, and estimates were combined across these datasets according to Rubin’s rule [16]. Statistical significance was set at an alpha level of 0.05. All statistical analyses were performed using R Version 4.3.3 (*R* Foundation for Statistical Computing, Vienna, Austria).

## 3. Results

Among all participants, 5.9% developed incident Type 2 diabetes. Table 1 presents the characteristics of the study participants; the average age was 56.5 years, with males comprising 39.3%. Of the participants, 49.0% completed high school, and 54.8% were employed. Regarding lifestyle habits, 64.8% were nonsmokers, and 38.0% were moderate alcohol consumers, consuming 1–22 g/day of ethanol. Notably, 37.6% reported sleeping < 6 h nightly; however, a significant majority (87.8%) engaged in > 2.5 h of exercise weekly. Mental stress was reported by 20.6% of the participants, with an average daily caloric intake of 1580.1 kcal. Most participants were of normal weight, with 75.3% having BMIs between 18.5 and 24.9 kg/m^2^ and 49.4% watching TV for < 3 h daily.

**TABLE 1:** Characteristics of study participants (*n* = 23,604).

	Original data	Multiple imputation
Age (years)	56.5 ± 9.5	56.5 ± 9.5
Sex		
Male	9284 (39.3)	(39.3)
Educational attainment		
Elementary school	838 (3.7)	(3.8)
Junior high school	7539 (33.7)	(33.8)
High school	10,972 (49.0)	(48.9)
College education or higher	3027 (13.5)	(13.5)
Missing	1228	
Paid job		
Present	12,488 (54.8)	(54.6)
Absent	10,300 (45.2)	(45.4)
Missing	816	
Smoking habits		
Never smokers	14,162 (64.8)	(66.2)
Former smokers	2546 (11.7)	(11.2)
Current smokers	5133 (23.5)	(22.6)
Missing	1763	
Drinking habits		
Never drinkers	11,249 (53.6)	(54.4)
Former drinkers	618 (2.9)	(2.9)
Current drinkers		
Of ethanol at 1–22 g/day	7972 (38.0)	(37.4)
Of ethanol at ≥ 23 g/day	1151 (5.5)	(5.3)
Missing	2614	
Daily sleep duration		
< 6 h/day	8553 (37.6)	(37.5)
6–6.9 h/day	4363 (19.2)	(19.2)
7–7.9 h/day	992 (4.4)	(4.4)
8–8.9 h/day	7198 (31.6)	(31.7)
≥ 9 h/day	1637 (7.2)	(7.2)
Missing	861	
Exercise		
< 2.5 h/week	2769 (12.2)	(12.3)
≥ 2.5 h/week	19,878 (87.8)	(87.7)
Missing	957	
Perceived mental stress		
Frequent	4497 (20.6)	(20.5)
Occasional	13,883 (63.5)	(63.5)
Seldom	3487 (15.9)	(15.9)
Missing	1737	
Average daily calorie intake (kcal)	1580.1 ± 457.5	1598.5 ± 480.1
Missing	4398	
Body mass index		
< 18.5 kg/m^2^	1191 (5.3)	(5.3)
18.5–24.9 kg/m^2^	16,989 (75.3)	(75.2)
25.0–29.9 kg/m^2^	4049 (18.0)	(18.0)
≥ 30 kg/m^2^	318 (1.4)	(1.4)
Missing	1057	
Average daily TV viewing time		
< 3 h/day	11,374 (49.4)	(49.4)
3–4.9 h/day	9098 (39.5)	(39.5)
5–6.9 h/day	2153 (9.3)	(9.4)
≥ 7 h/day	407 (1.8)	(1.8)
Missing	572	

Values are expressed as mean ± standard deviation or *n* (%). Percentages may not total 100% due to rounding.

Table 2 shows the association between BMI and incident Type 2 diabetes. After adjusting for potential confounders in Model 3, the ORs and 95% CIs for Type 2 diabetes incidence were 0.71 (0.53–0.95) for individuals with BMI < 18.5 kg/m^2^, 1.76 (1.54–2.01) for those with BMI between 25.0 and 29.9 kg/m^2^, and 3.40 (2.45–4.37) for individuals with BMI ≥ 30 kg/m^2^, compared to the reference group (BMI 18.5–24.9 kg/m^2^). This indicated a significant trend of increasing diabetes risk with higher BMI categories (*p* for trend < 0.001).

**TABLE 2:** The association between body mass index and incident diabetes (*n* = 23,604).

	Body mass index (kg/m^2^)				*p* for trend
	< 18.5	18.5–24.9	25.0–29.9	≥ 30	
Incidence rate (/1000 person)	44.9	52.7	84	145.4	
OR (95% CI)^1^	0.73 (0.55, 0.98)^∗^	Ref	1.78 (1.57, 2.03)^∗^	3.54 (2.56, 4.90)^∗^	< 0.001
OR (95% CI)^2^	0.71 (0.53, 0.95)^∗^	Ref	1.79 (1.57, 2.04)^∗^	3.53 (2.54, 4.90)^∗^	< 0.001
OR (95% CI)^3^	0.71 (0.53, 0.95)^∗^	Ref	1.76 (1.54, 2.01)^∗^	3.40 (2.45, 4.37)^∗^	< 0.001

^∗^ *p* < 0.05. OR, odds ratio; CI, confidence interval.

^1^Adjusted for age and sex.

^2^Further adjusted for educational attainment, paid job, smoking habits, drinking habits, daily sleep duration, perceived mental stress, hours of exercise, and average daily calorie intake.

^3^Further adjusted for average daily television viewing time.

Table 3 examines the association between the average daily TV viewing time and incident Type 2 diabetes. The ORs and 95% CIs for the incidence of Type 2 diabetes were 1.23 (1.09–1.40) for individuals watching TV 3.0–4.9 h/day, 1.37 (1.14–1.66) for those watching 5.0–6.9 h/day, and 1.75 (1.25–2.44) for individuals watching ≥ 7.0 h/day, compared to the reference group (average daily TV viewing time < 3.0 h/day). These findings demonstrate a significant trend of increased Type 2 diabetes risk with higher TV viewing time categories (*p* for trend < 0.001).

**TABLE 3:** The association between average daily television viewing time and incident diabetes (*n* = 23,604).

	Television viewing time (hours/day)				*p* for trend
	< 3.0	3.0–4.9	5.0–6.9	≥ 7.0	
Incidence rate (/1000 person)	48.6	64.9	82.2	109.8	
OR (95% CI)^1^	Ref	1.28 (1.14, 1.45)^∗^	1.53 (1.27, 1.83)^∗^	2.09 (1.51, 2.89)^∗^	< 0.001
OR (95% CI)^2^	Ref	1.27 (1.12, 1.44)^∗^	1.45 (1.20, 1.75)^∗^	1.89 (1.35, 2.63)^∗^	< 0.001
OR (95% CI)^3^	Ref	1.23 (1.09, 1.40)^∗^	1.37 (1.14, 1.66)^∗^	1.75 (1.25, 2.44)^∗^	< 0.001

^∗^ *p* < 0.05. OR, odds ratio; CI, confidence interval.

^1^Adjusted for age and sex.

^2^Further adjusted for educational attainment, paid job, smoking habits, drinking habits, daily sleep duration, perceived mental stress, hours of exercise, and average daily calorie intake.

^3^Further adjusted for television viewing time.

Table 4 details the ORs of incident Type 2 diabetes for the joint exposure of BMI and average daily TV viewing time. For individuals with a BMI < 18.5 kg/m^2^, TV viewing time did not significantly alter the risk of developing Type 2 diabetes. However, participants with a BMI between 18.5 and 24.9 kg/m^2^ experienced a progressive increase in diabetes risk with longer durations of TV viewing. Specifically, those watching TV for ≥ 7 h exhibited a significantly increased risk (OR: 1.76, 95% CI: 1.14–2.71) compared to the reference group, which included individuals who viewed TV < 3 h daily. Moreover, for individuals with a BMI ≥ 30, viewing TV for ≥ 7 h was associated with a twentyfold increase in the risk of diabetes (OR: 21.42, 95% CI: 7.23–63.49), with a *p* value for interaction = 0.02.

**TABLE 4:** The odds ratios of incident diabetes for the joint exposure of body mass index and average daily television viewing time (*n* = 23,604).

TV viewing time (hours/day)	Body mass index (kg/m^2^)							
	< 18.5		18.5–24.9		25.0–29.9		≥ 30	
	Incidence rate (/1000 person)	OR (95% CI)	Incidence rate (/1000 person)	OR (95% CI)	Incidence rate (/1000 person)	OR (95% CI)	Incidence rate (/1000 person)	OR (95% CI)
< 3	32.9	0.65 (0.41, 1.05)	41.8	Ref	82.4	2.21 (1.80, 2.70)^∗^	95.7	2.62 (1.42, 4.83)^∗^
3–4.9	59.2	1.14 (0.76, 1.73)	59.5	1.33 (1.14, 1.54)^∗^	82	2.07 (1.69, 2.54)^∗^	134.4	4.06 (2.40, 6.85)^∗^
5–6.9	43	0.67 (0.27, 1.66)	78.6	1.56 (1.25, 1.96)^∗^	90.9	2.10 (1.49, 2.97)^∗^	206.9	5.79 (2.85, 11.75)^∗^
≥ 7	73.1	1.38 (0.32, 5.95)	91.4	1.76 (1.14, 2.71)^∗^	122.5	2.37 (1.15, 4.89)^∗^	462.3	21.42 (7.23, 63.49)^∗^

^∗^ *p* < 0.05. OR, odds ratio; CI, confidence interval adjusted for age, sex, educational attainment, paid job, smoking habits, drinking habits, daily sleep duration, perceived mental stress, hours of exercise, and average daily calorie intake.

## 4. Discussion

This prospective population‐based cohort study conducted within the Japanese population found that both overweight/obesity and prolonged TV viewing times were independently associated with an increased risk of developing Type 2 diabetes. Notably, individuals who were overweight or obese and engaged in extended TV viewing exhibited a significantly increased risk, highlighting the statistically significant interaction between these factors.

These findings align with existing research, as a meta‐analysis of prospective studies demonstrated a substantial increase in the risk of Type 2 diabetes in overweight and obese individuals. Compared to normal weight, the pooled relative risks (RRs) of Type 2 diabetes were 2.24 (95% CI: 1.95–2.56, based on 47 reports) for overweight individuals and 4.56 (95% CI: 3.69–5.64, based on 43 reports) for obese individuals [6]. In this study, individuals with a BMI < 18.5 kg/m^2^ exhibited a significantly lower OR for Type 2 diabetes incidence. However, the referenced study reported no significant association between being underweight (RR: 0.93, 95% CI: 0.75–1.15, based on 12 papers) and Type 2 diabetes risk. Despite these findings, two large‐scale studies [17, 18] included indicated a potentially reduced Type 2 diabetes risk among underweight individuals compared with those with normal weight. The China Kadoorie Biobank study, which enrolled 512,891 adults aged 30–79 from 10 locations across China between 2004 and 2008, observed over a follow‐up period of 9.2 years that underweight individuals had a lower risk of developing certain conditions, with a hazard ratio (HR) of 0.64 (95% CI: 0.52–0.77) and 0.61 (95% CI: 0.53–0.71) for males and females, respectively [17]. Among 12,672 white and African American males and females aged 45–64 years who participated in the Atherosclerosis Risk in Communities Study’s first visit (1987–1989) and reexamined at three subsequent follow‐up examinations, the associations with incident diabetes were analyzed using Cox proportional hazard models. Over the 9‐year follow‐up, underweight individuals were found to have a lower risk of developing certain conditions. Specifically, underweight white women exhibited an HR of 0.59 (95% CI: 0.38–0.94), indicating a decreased risk of developing diabetes [18].

The association between TV viewing time and Type 2 diabetes risk has also been corroborated by previous prospective cohort studies [19–24]. Supporting these findings, a meta‐analysis of four prospective studies involving 175,938 participants reported a 20% increased risk of Type 2 diabetes for every additional 2 h of TV viewing per day [7]. Our research enhances the current knowledge by detailing the combined risk associated with obesity and excessive TV viewing, a prevalent form of sedentary behavior, illustrating the significant impact of their interaction on health outcomes. The substantially higher risk in individuals with a BMI ≥ 30 kg/m^2^ who spend extended periods watching TV highlights the urgent need for comprehensive health interventions. Such interventions should address both weight management and sedentary behavior reduction. Additionally, the gradual rise in diabetes risk with increased TV viewing time in individuals with BMIs between 18.5 and 24.9 kg/m^2^ sheds light on the impact that sedentary behavior can have on health. This indicates that public health guidelines should aim to curtail sedentary leisure activities as a broader strategy for diabetes prevention.

The connection between overweight or obesity and Type 2 diabetes risk in individuals with prolonged TV viewing is not fully understood; however, potential pathways have been identified. Obesity, which increases Type 2 diabetes risk, involves the release of higher levels of nonesterified fatty acids, glycerol, hormones, proinflammatory cytokines, and other substances from adipose tissues, contributing to insulin resistance development [25]. Sedentary lifestyles, including extensive TV viewing, are speculated to decrease skeletal muscle glucose uptake owing to impaired glucose transporter Type 4 translocation and reduced lipoprotein lipase activity in skeletal muscles [26]. Furthermore, prolonged TV viewing was associated with low energy expenditure and consumption of unhealthy, calorie‐dense foods, which led to weight gain and obesity [8]. Mediation analysis suggests that BMI may play a role in mediating the relationship between TV viewing time and Type 2 diabetes risk [27]. Conversely, overweight or obese adults exhibit lower physical activity levels and longer TV viewing times [28]. This complex interplay between lifestyle choices and biological mechanisms underscores the importance of adopting more active lifestyles to mitigate the diabetes risks.

The strength of our analysis lies in its prospective, population‐based study design, with participants from all over Japan, providing a solid framework for our investigation. However, this study has some limitations that should be considered when interpreting the results. First, weight and height were self‐reported rather than directly measured. However, a validation study of a Japanese population showed a strong correlation between self‐reported and measured weight and height [29]. Second, TV viewing time was self‐reported, which raises concerns about misclassification. However, a recent study of Japan confirmed the reliability of self‐reported TV viewing time, with interclass correlation coefficients of 0.76 on workdays, 0.79 on nonworkdays, and 0.82 across an entire week [30]. In addition, TV viewing time was assessed as an average daily duration over the past year and was not differentiated by weekdays, weekends, or holidays, which may have introduced recall bias and obscured short‐term variability in viewing patterns. Furthermore, TV viewing time was used as a proxy for sedentary screen‐based behavior, and information on other forms of screen time, such as computers, mobile devices, or video games, was not available. Therefore, total screen time could not be comprehensively assessed. Third, detailed dietary behaviors such as junk food consumption, snacking frequency, or sugar intake were not comprehensively assessed in the baseline survey and therefore could not be included in the analyses, leaving the possibility of residual confounding related to unmeasured dietary factors. Fourth, information on family history of diabetes, baseline glycemic status such as fasting glucose or HbA1c levels, comorbid conditions including hypertension and dyslipidemia, and medication use was not available in the baseline survey and therefore could not be adjusted for in the analyses. As a result, residual confounding related to these unmeasured factors cannot be completely excluded. Fifth, the prevalence of obesity defined as BMI ≥ 30 kg/m^2^ was low in this cohort, reflecting the body weight distribution of the Japanese population. Therefore, the generalizability of our findings to populations with a higher prevalence of obesity may be limited. Sixth, perceived mental stress was assessed using a self‐reported measure, which may not fully capture chronic or physiological stress exposure. As a result, misclassification of stress levels is possible. Seventh, we used self‐reported, physician‐diagnosed diabetes cases, which may have led to misclassifications. Nonetheless, an earlier study on the same cohort reported moderate sensitivity and high specificity for self‐reported diabetes in evaluating diabetes based on plasma glucose levels and treatment with hypoglycemic medications [14]. A two‐point assessment of Type 2 diabetes at baseline and 5 years later in the present study is a weaker design than yearly assessments because diabetes occurrence cannot be fully captured during follow‐up.

In conclusion, our study showed the importance of addressing both obesity and sedentary behavior to mitigate the risk of Type 2 diabetes. By highlighting the combined risk associated with high BMI and extensive TV viewing, our findings support the development of multifaceted public health strategies aimed at promoting physical activity and maintaining a healthy weight as integral components of diabetes prevention.

## Author Contributions

Shigekazu Ukawa: formal analysis and writing of the original draft. Wenjing Zhao, Satoyo Ikehara, Kokoro Shirai, Hiroyasu Iso, and Akiko Tamakoshi: review and editing.

## Funding

This study has been supported by Grants‐in‐Aid for Scientific Research from the Ministry of Education, Culture, Sports, Science and Technology of Japan (MEXT) (MonbuKagaku‐sho); Grants‐in‐Aid for Scientific Research on Priority Areas of Cancer; and Grants‐in‐Aid for Scientific Research on Priority Areas of Cancer Epidemiology from MEXT (Nos. 61010076, 62010074, 63010074, 1010068, 2151065, 3151064, 4151063, 5151069, 6279102, 11181101, 17015022, 18014011, 20014026, 20390156, and 26293138), and JSPS KAKENHI No.16H06277. This research was also supported by Grant‐in‐Aid from the Ministry of Health, Labour and Welfare, Health and Labor Sciences research grants, Japan (Comprehensive Research on Cardiovascular Disease and Life‐Style Related Diseases: H20–Junkankitou [Seishuu]–Ippan–013; H23–Junkankitou [Seishuu]–Ippan–005); an Intramural Research Fund (22‐4‐5) for Cardiovascular Diseases of National Cerebral and Cardiovascular Center; Comprehensive Research on Cardiovascular Diseases and Life‐Style Related Diseases (H26‐Junkankitou [Seisaku]‐Ippan‐001) and H29–Junkankitou [Seishuu]–Ippan–003 and 20FA1002.

## Disclosure

All the authors have read and approved the final manuscript.

## Ethics Statement

Individual informed consent before participation in the study was obtained in 36 of the 45 study areas, with written informed consent in 35 areas and oral consent in 1 area. In the remaining 9 areas, group consent was obtained from the respective area leaders. This study was approved by the Ethics Committee of the Faculty of Medicine, Hokkaido University (approval no.: 14‐044) and was performed in accordance with the principles of the Declaration of Helsinki.

## Consent

In most regions, informed consent was obtained individually and directly from cohort members. In other areas, informed consent was obtained at the community level after the purpose of the study was explained, and the confidentiality of the data was explained to community leaders and mayors.

## Conflicts of Interest

The authors declare no conflicts of interest.

## Data Availability

The data presented in this study are not publicly available due to privacy and ethical restrictions.
